# Oxidative Stability and Kinetics of Oxidation of Rosehip, Sunflower, Olive and Jojoba Oils

**DOI:** 10.3390/antiox15050646

**Published:** 2026-05-20

**Authors:** Carmen Fagoaga, Angela Moreno, Nayara Fernández-Julián, Gloria Castellano

**Affiliations:** 1 Research Group Structure-Bioactivity Relationship of Organic Compounds, Facultad de Veterinaria y Ciencias Experimentales, Universidad Católica de Valencia San Vicente Mártir, 46001 Valencia, Spain; carmen.fagoaga@ucv.es (C.F.); nayara.fernandez@mail.ucv.es (N.F.-J.); 2 Centro de Investigación Traslacional San Alberto Magno (CITSAM), Universidad Católica de Valencia San Vicente Mártir, 46001 Valencia, Spain

**Keywords:** oxidative stability, antioxidant capacity, BQC-Redox System (BRS), Rancimat, jojoba, rosehip, olive, sunflower, oils

## Abstract

Lipid oxidation affects the quality and functionality of vegetable oils, and its progression depends largely on fatty acid composition and antioxidant content. Oxidation kinetics provide essential information about oxidative resistance in oils. The determination of activation parameters allows for the evaluation of oxidation susceptibility under thermal stress. Oxidative stability and oxidation kinetics at different temperatures of rosehip, sunflower, olive and jojoba oils were studied using both Rancimat and BQC-Redox System methods, enabling the calculation of kinetic constants and thermodynamic activation parameters for the process. BRS measurements showed an increase in total antioxidant capacity (TAC) with temperature in all samples, with olive oil presenting the highest TAC and jojoba the lowest at 298 K, while rosehip oil showed the lowest TAC at 373 K. Kinetic analysis revealed negative ΔS*^#^* values, indicating the formation of ordered transition states, and similar activation energies (ΔG*^#^* ≈ 56–58 kJ/mol), although jojoba displayed the highest ΔH*^#^* and ΔG*^#^*. Rancimat analysis at 373 K showed clear differences in oxidative stability: jojoba oil had the longest induction period, followed by olive, sunflower, and rosehip. These results correlated with PUFA levels. Principal component analysis (PCA) confirmed strong associations between induction period, fatty-acid composition, and kinetic parameters, demonstrating good agreement between the two analytical methods.

## 1. Introduction

Vegetable oils are essential sources of dietary energy and are widely used in food and cosmetic applications, with increasing global demand driven by their health benefits and functional properties [1,2,3]. In the cosmetic sector, the growing preference for sustainable and natural ingredients has promoted the use of plant-derived oils and extracts as antioxidants, regenerators, and moisturizers, reducing reliance on synthetic irritants [4]. Lipophilic extracts, particularly from aromatic plants, are rich in phenolic compounds derived from secondary metabolism [5,6], while cold-pressed oils are generally considered safe for topical use due to their low incidence of irritation or allergic reactions [7].

Despite these advantages, the quality of vegetable oils is strongly limited by lipid oxidation, a temperature-dependent process that produces volatile compounds responsible for rancidity and the loss of nutritional and functional value [8,9]. Oxidation is influenced by factors such as light, heat, enzymes, and microorganisms, and proceeds via auto-, photo-, thermal, and enzymatic pathways involving free radical formation. Auto-oxidation is the predominant mechanism, generating hydroperoxides and secondary products such as alcohols, carbonyls, and carboxylic acids [10], while enzymatic oxidation, mainly catalyzed by lipoxygenases, preferentially affects polyunsaturated fatty acids (PUFAs) [11]. Consequently, oxidative stability is a key parameter determining oil shelf life and functionality, requiring reliable analytical indicators to monitor oxidation kinetics [12]. Natural antioxidants, including polyphenols, tocopherols, and phytosterols, play a crucial role in inhibiting oxidation [13], whereas susceptibility largely depends on fatty acid composition, particularly PUFA content [14,15,16]. Oils such as sunflower (*Helianthus annuus*) are widely valued due to their thermo-oxidative behavior in both food and cosmetic applications [17,18].

In this context, the present study aimed to evaluate the oxidative stability, temperature dependence, oxidation kinetics, and antioxidant capacity of four oils with different compositions and applications: jojoba and rosehip oils (cosmetic use), and olive and sunflower oils (food use). This research is of particular significance as it provides an integrated and quantitative framework to better understand the mechanisms governing lipid oxidation, thereby contributing to the optimization of oil selection and processing in both food and cosmetic industries. Oxidative stability was assessed in pure samples using two complementary techniques: the Rancimat method and the BQC-Redox System (BRS). The Rancimat method determines the induction time, a widely accepted measure of resistance to oxidation under accelerated conditions, based on the conductometric detection of volatile oxidation products generated at elevated temperatures and continuous airflow [19]. In contrast, the BRS provides information on total antioxidant capacity (TAC) through an electrochemical approach, measuring the current produced by the oxidation of antioxidant compounds under an applied potential, offering a rapid and reproducible analysis [20].

The BRS method (BQC-Redox System) is a portable device that utilizes electrochemical technology. This product has been developed for the rapid and reproducible measurement of antioxidant capacity of different types of samples. In this method, the antioxidant components of the samples are oxidized by applying a variable electrical potential. Electrons are released from antioxidant species and detected as an electrical current. This method is important because of its speed, simplicity and versatility, as it allows different types of samples to be analyzed, whether extracts, oils, foods or cosmetics, using small volumes [20].

The combined use of both techniques enhances analytical reliability by providing complementary information on oxidative resistance and antioxidant behavior. Additionally, temperature-dependent measurements enable the calculation of kinetic constants and activation thermodynamic parameters (ΔH^#^, ΔS^#^, ΔG^#^), offering quantitative insight into oxidation mechanisms [21]. The results derived from this approach are expected to provide valuable and insightful information on the relationship between composition and oxidative behavior, with potential implications for improving the stability and application of vegetable oils.

The selected oils differ markedly in composition. Jojoba oil, derived from *Simmondsia chinensis*, consists mainly of wax esters (≈98%) and is often described as a “liquid wax,” exhibiting high oxidative resistance due to its monounsaturated structure [22,23]. Rosehip oil (*Rosa canina*) is rich in MUFAs and PUFAs, particularly α-linolenic and linoleic acids, and contains tocopherols with strong antioxidant activity, although its high unsaturation reduces stability [24,25,26]. Sunflower oil (*Helianthus annuus* L.) is characterized by high linoleic and oleic acid content and significant vitamin E levels, contributing to its nutritional value [27,28]. Olive oil (*Olea europaea* L.) is predominantly composed of oleic acid-rich triacylglycerols and contains minor bioactive compounds such as phenolics and tocopherols with recognized health benefits, forming a key component of the Mediterranean diet [29,30,31].

Finally, results were interpreted in relation to oil composition, and principal component analysis (PCA) was applied to explore relationships among compositional variables, oxidative stability indices, and kinetic parameters. This integrated approach provides a comprehensive understanding of the factors governing lipid oxidation and supports the rational selection of oils for food and cosmetic applications where oxidative stability is a critical quality parameter.

## 2. Materials and Methods

### 2.1. Oils Samples Used

Commercial edible and cosmetic oils were purchased from a local market and were stored at room temperature under dark conditions until the analysis.

Extra virgin olive oil was selected because it is one of the few oils that is consumed without refining [32], and refined sunflower oil was selected due to its high global consumption, economic significance and widespread use in the food industry.

Cosmetic oils (jojoba and rosehip) were obtained through cold pressing, thereby preserving valuable phytochemical compounds that contribute to their functional properties. Both were chosen for their different fatty acid profiles.

Data related to the origin of the four oil samples are shown below and the composition has been provided by the manufacturer. It is shown in Table 1.

- Cold-pressed jojoba oil: *Simmondsia chinensis* seed oil. Laboratorio Cosmética Natural CELEPLAME-S.L. Moncada, Valencia, Spain. Batch number: AYO24O.
- Cold-pressed rosehip oil: *Rose canina* seed oil. Laboratorio Cosmética Natural CELEPLAME-S.L. Moncada, Valencia, Spain. Batch number: ARM25E.
- Refined sunflower oil: *Heliannthus annus* seed oil. Manufacturer: Koipe Sol, Alcolea, Córdoba, Spain. Batch number: L54068C.
- Extra virgin olive oil: *Olea europea* fruit oil. Manufacturer: Oleo Masía S.A. Dos Hermanas, Sevilla, Spain. Batch number: L24200A2.

### 2.2. Basics of the BRS Test for Oil Oxidation

The BRS (BQC-Redox System) developed by Bioquochem ( Oviedo, Spain) is based on a voltammetric electrochemical method designed to evaluate the total antioxidant capacity of a sample through the direct oxidation of reducing species present in the matrix. During the analysis, a potential sweep is applied to the electrode, which induces the oxidation of antioxidant compounds once their characteristic redox potential is reached. In this process, antioxidant molecules donate electrons to the electrode and are converted into their oxidized forms, generating an electrical current proportional to the number of electrons transferred. The integration of this current allows for the calculation of the total electrical charge associated with the oxidation reactions. This parameter is used as an indicator of the overall antioxidant capacity of the sample.

Total antioxidant capacity is expressed as TAC (BRS values), TEAC (Trolox Equivalent Antioxidant Capacity, measured in μM Trolox equivalents), and CEAC (Catechin Equivalent Antioxidant Capacity, expressed in μM equivalents of the antioxidant standard ascorbic acid). These metrics enable comparison of the antioxidant activity of different samples relative to reference compounds [33]. The electrical current detected signal from the oxidized sample is recorded by the system, providing the results through the software [34].

#### 2.2.1. Pure Oil Samples Preparation

First, 0.02 g of each oil was weighed separately and mixed with 400 μL of a 1:1 methanol/BRS electrolyte mixture in Eppendorf tubes. The samples were sonicated for 10 min under maximum vibration in a sonicator (OVAN model) and then vortexed to ensure the samples were homogeneous.

A blank was prepared using a mixture of methanol and BRS electrolyte 1:1.

#### 2.2.2. Measurement of the Antioxidant Capacity of Oils Using the BRS Test

The total antioxidant capacity of oils was determined using the BRS equipment. For each sample, the TAC values were obtained by performing the measurements in triplicate. The BRS device was connected to a mobile device with the application installed for recording measurement data via Bluetooth, which allowed for automatic data visualization and storage.

Initially, the device was calibrated according to the manufacturer’s instructions. Each temperature was stabilized for 10 min using a thermostatic bath.

A blank measurement was then performed, and for each sample and measurement a new strip was used.

Subsequently, 20 μL of each prepared oil sample was applied onto a test strip and inserted into the device for analysis, and the measurement was performed for about 4–5 s. Once the sample had been applied to a new test strip and inserted into the device, the measurement was carried out as quickly as possible and accurately immediately after application.

TAC measurements were carried out at different temperatures ranging from 298 to 373 K. All measurements were performed in triplicate for each temperature, and the reported values correspond to the mean of three independent determinations.

#### 2.2.3. Analysis of Reaction Activity Kinetics

TAC data were obtained using the BRS test and were used in kinetic analysis that was performed according to the Eyring equation derived from transition state theory (TST). This theory relates the activation energy to the energy difference between the transition state and the reactants, forming a high-energy activated complex [35]. The Eyring equation enables direct determination of thermodynamic parameters associated with the transition state, including the rate constant (k), activation enthalpy (ΔH^#^), activation entropy (ΔS^#^), and Gibbs free energy at 298 K (ΔG^#^) [36].

Measurements were performed independently in triplicate at each temperature. For every temperature investigated, mean values were calculated from the corresponding experimental measurements and used as representative experimental data points for kinetic regression analysis. Thus, kinetic calculations were based on experimentally obtained values at each temperature, while averaging was applied only to replicate measurements performed under identical experimental conditions. The natural logarithms of TAC/T values were plotted against the reciprocal temperature (1/T, K^−1^) to obtain linear relationships from which k_298K_, ΔH^#^, ΔS^#^, and ΔG^#^ were calculated according to the Eyring model:

In this study, the reaction rate constant (k) was estimated using total antioxidant capacity (TAC) due to the short reaction times. TAC was measured by the reagent-free BRS electrochemical method (Bioquochem), based on direct electron transfer between antioxidants and the electrode. Under initial conditions (very short time intervals), TAC (or TAC/t) is proportional to the initial reaction rate and thus to an apparent value of k. As all measurements were performed at the same acquisition time across temperatures, TAC was considered proportional to k for comparative kinetic analysis.

This approach does not affect the activation enthalpy (ΔH^#^) obtained from the Eyring plot slope, although it introduces a constant offset in the intercept; since this effect is systematic, the activation entropy (ΔS^#^) values remain suitable for comparison among samples.

$$\begin{array}{l} \;\;\;\;\;\;\;\;\;\;\;\;\;\;\;\;\;\;\;\;\;\;\;\;\;\;\;\;\;\;\;\;\;\ln\left(\frac{k}{T}\right)=-\left(\frac{\Delta H^{\#}}{R}\right)\frac{1}{T}+\left(\frac{{\Delta S}^{\#}}{R}\right)+\ln\left(\frac{k_{B}}{h}\right)\\ y=\ln\left(\frac{k}{T}\right)\;\;\;\;\;\;\;\;\;\;m=-\left(\frac{\Delta H^{\#}}{R}\right)\;\;\;\;\;\;\;\;\;\;\;\;x=\frac{1}{T}\;\;\;\;\;\;\;\;\;\;\;b=\left(\frac{{\Delta S}^{\#}}{R}\right)+\ln\left(\frac{k_{B}}{h}\right) \end{array}$$

R = Gas constant (8.314 J/mol × K)

k_B_ = Boltzmann constant (1.381 × 10^−23^ J/K)

h = Planck constant (6.626 × 10^−3^^4^ J × s)

The values of ∆G^#^ and k_298K_ were calculated using the following equations:

$$\Delta G^{\#}=\Delta H^{\#}-T\Delta S^{\#}$$

$$k=\frac{k_{B}T}{h}\exp(-\frac{{\Delta G}^{\#}}{\mathrm{RT}})$$

Moreover, data of k_373K_ was obtained starting from k_298K_ and the Arrhenius equation.

$$\ln\left(\frac{k_{2}}{k_{1}}\right)=\frac{\mathrm{Ea}}{R}\left(\frac{1}{T_{1}}-\frac{1}{T_{2}}\right)$$

where T_1_ = 298 K. T_2_ = 373 K.

**Figure 1:**
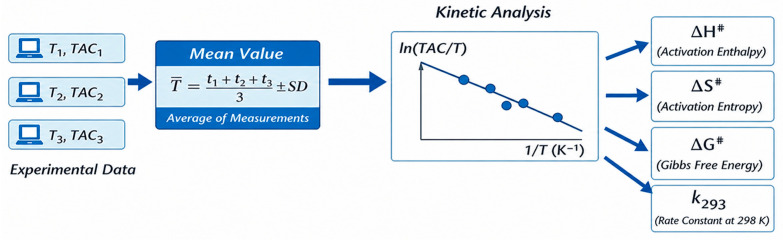
Schematic representation of the experimental workflow and kinetic data treatment used for the calculation of activation parameters according to the Eyring equation.

The experimental design previously described is presented in Figure 1.

### 2.3. Fundamentals of the Rancimat Method for Oil Oxidation Analysis

In this study, the Rancimat model 892 was used (Metrohm AG, Herisau, Switzerland), which automatically records the variation in conductivity over time, generating oxidation curves that are represented in graphs using StabNet 1.1 software. The principle of the method consists of subjecting the sample to be analyzed to a constant and elevated temperature under a controlled air flow. In this way, the sample oxidizes, generating volatile secondary products, which the air stream carries to a measuring vessel, where they are absorbed by a solution of deionized distilled water, and the electrical conductivity is recorded. The IP is the time at which the conductivity curve, initially stable, begins to rise sharply, leading to the accumulation of volatile compounds [19].

#### 2.3.1. Pure Oil Sample Preparation

Once all the parameters had been established, the oil samples were prepared for oxidation. Three grams of each pure oil sample were weighed using a precision balance and placed in the equipment’s reaction tubes. The samples were analyzed in quadruplicate in order to minimize variability and improve reproducibility between samples.

#### 2.3.2. Measurement of the Oxidative Stability of Oils Using the Rancimat Method

According to previous oil oxidation tests conducted using the Rancimat method [37], a temperature of 373 K and a constant air flow of 20 L/h were established as experimental conditions to measure the oxidative stability of the oils.

All measurements were performed in quadruplicate and the results were recorded on a computer and analyzed using the software package Stab-Net 1.1 provided by the manufacturer.

#### 2.3.3. Assembly of the Rancimat

First, 60 mL of deionized distilled water was prepared and introduced into the eight measuring vessels, ensuring that the initial conductivity was less than 2.4 μS/cm. These vessels were connected to the oil reaction tubes, allowing the volatile products generated during the oxidation time to be carried into the water. The time required for a sharp increase in water conductivity was calculated by the instrument’s software package (StabNet 1.1) and corresponds to the induction period in hours. The most stable oil samples presented the highest values of the induction period.

#### 2.3.4. Statistical Analysis

All measurements were performed using independent replicates to ensure experimental reliability. TAC measurements obtained using the BQC-Redox System (BRS) were carried out in triplicate at each investigated temperature. Mean values and standard deviations were calculated from the experimental measurements. The results were presented both as direct plots of TAC versus temperature and through kinetic analysis based on the Eyring model. Mean values corresponding to each temperature were used as representative experimental data points for regression analysis.

Rancimat measurements were performed in quadruplicate at 373 K, and induction times were expressed as mean values ± standard deviation of the four independent determinations.

Principal component analysis (PCA) was applied as a multivariate statistical tool to explore relationships among oxidative stability parameters, kinetic variables, and compositional characteristics of the oils. Prior to PCA, variables were standardized to eliminate scale effects, and the analysis was performed using the correlation matrix. The rotation method used was Varimax with Kaiser normalization. Therefore, PCA was used exclusively as an exploratory and descriptive tool due to the limited sample size (n = 4). To ensure statistical robustness, the analysis was complemented by linear regression models evaluating the relationship between fatty acid composition and antioxidant capacity. PCA was made with SPSS (vs. 21.0, IBM Corp., Chicago, IL, USA) software.

## 3. Results and Discussion

### 3.1. Evaluation of Temperature Influence in Total Antioxidant Capacity (TAC)

At the mechanistic level, the electrochemical reaction occurs at the electrode solution interface and involves several steps: diffusion of antioxidants toward the electrode surface, entry into the electrochemical double layer, and heterogeneous electron transfer. During this transfer, a transition state or activated complex is formed in which the electron is partially shared between the reducing species and the electrode before complete oxidation occurs: Reductant + Electrode → [Reductant^δ+^⋯e^−^⋯Electrode]^#^ [35,36]. The electrolyte used in the system plays an essential role by providing ionic conductivity, stabilizing the pH and ionic strength of the medium, and minimizing electrical migration effects. This ensures that the transport of species toward the electrode occurs predominantly by diffusion and guarantees reproducible electrochemical conditions during the measurement.

In comparative studies of antioxidants, a mixture of electrolyte and methanol can be used, provided that all samples and the blank have exactly the same composition, pH, and solvent proportion, since methanol does not undergo oxidation within the applied potential range. Using the same mixture as the blank corrects for solvent effects on background current, conductivity, and the electrochemical double layer, ensuring that the measured signal primarily reflects the oxidation of the antioxidants. In this way, although the medium may slightly alter the absolute response, the relative differences between antioxidants remain comparable, allowing for a reliable assessment of their antioxidant capacity in comparative studies.

**Figure 2:**
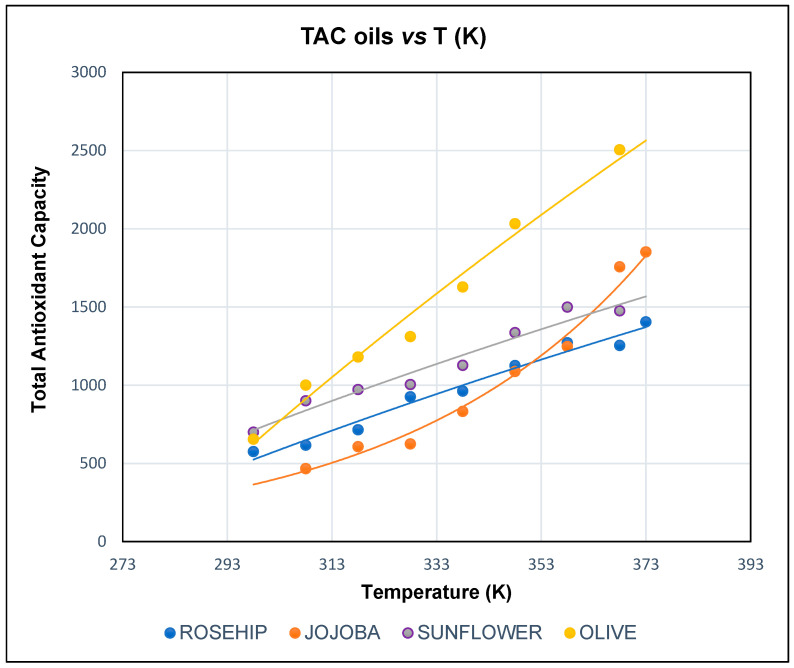
Variation in TAC (BRS values) as a function of temperature (K) in pure rosehip, jojoba, sunflower and olive oils.

The results of TAC are presented in Figure 2. Olive, sunflower and rosehip oils exhibited an upward lineal behavior of their TAC with increasing temperature; however, jojoba oil showed an exponential behavior. Olive oil showed the highest values in all the tested temperatures above 300 K, and jojoba oil showed the lowest values for tested temperatures under 340 K.

Figure 2 shows that when the temperature is 298 K, jojoba oil has the lowest value, but at 373 K, rosehip oil has the lowest TAC value.

Moreover, in Table 2, the results show that sunflower and rosehip oils have straight lines with a lower slope and similar values, indicating that their antioxidant capacity varies less with temperature. Both olive oil, which has a steeper slope, and jojoba oil, with exponential behavior, show much greater variability. It is important to remember that the TAC measurements at each temperature were taken about 4–5 s after reaching these temperatures (very short periods at each temperature).

The results show that TAC improves with increasing temperature in all oils, in accordance the results obtained in a recent study showing that the highest antioxidant activity was observed at temperatures above 358 K. This can be explained by the fact that the increase in temperature produces greater fluidity of the lipid matrix, which favors the mobilization and availability of antioxidants that were previously confined within structures such as lipid vesicles, which impede the antioxidant ability to donate electrons to the radical, neutralizing it [38].

Therefore, temperature increased antioxidant activity (TAC) in all systems, promoting the mobility and action of bioactive compounds.

Remarkably, jojoba oil is the only sample whose behavior changes significantly with temperature, showing an exponential model. In all oils, antioxidant capacity increases with temperature, with the strongest effects observed in olive and jojoba oils. However, in the case of jojoba oil, its response shifts substantially above 340 K, rising from the lowest antioxidant capacity among the oils to the second highest, after olive oil. This observation suggests that important physical chemical processes that could be associated with compositional changes may occur in jojoba oil above this temperature threshold.

In the case of jojoba oil, the measurement at 298 K consistently showed anomalous values compared with the overall trend. This behavior may be related to the particular physicochemical properties of jojoba oil, which is mainly composed of long-chain monounsaturated wax esters and exhibits higher viscosity and different interfacial behavior at lower temperatures compared with the other oils. Similar isolated exclusions were applied for sunflower and olive oils at the temperatures indicated in Figure 2 when anomalous values were observed.

It is suggested that higher temperatures may enhance the fluidity of jojoba oil, leading to an exponential rise in TAC values, whereas the other oils, which are less viscous, exhibit a more linear increase.

Vegetable oils are available as refined or cold-pressed oils, which exhibit different losses of minor compounds during the industrial process. Typically, refined oils present higher losses of phenolic compounds, tocopherols, phytosterols, and carotenoids, becoming more prone to oxidation [39]. In contrast, cold-pressing does not involve heat or chemical processes, resulting in oils that maintain high contents of these antioxidant compounds. However, cold-pressed oils tend to contain more oxidizable compounds than refined oils [40]. Therefore, the quality of the oil should be considered holistically by simultaneously considering the industrial process, chemical composition, and oxidative stability. The total antioxidant capacity (TAC) of vegetable oils results from the integrated action of the complex mixture of antioxidants present in oils against oxidation reactions. Castelo-Branco et al. 2016 [41] assess the potential of TAC in refined and cold-pressed vegetable oils as an indicator of quality and oxidative stability, which ultimately determines shelf life.

### 3.2. Thermodynamic and Kinetic Analysis of TAC from Oils Samples

For the thermodynamic and kinetic analysis, the Eyring equation was applied, transforming the TAC values into ln (TAC/T(K)) *versus* 1/T(K), as can be seen in Table 3, which allowed for the thermodynamic parameters of the transition state to be calculated.

From a kinetic point of view, the parameters obtained using the Eyring equation (∆H^#^, ∆S^#^, ∆G^#^ and k_298K_) provide information on the antioxidant process taking place within the oils, assess whether the chemical reaction is exothermic or endothermic (ΔH^#^), and determine the degree of disorder in a system (ΔS^#^) [42]. The k_373K_ values were calculated using the Arrhenius equation from k_298K_. In all oils, the ∆S^#^ values are negative, meaning that the transition state is more ordered than the initial state. In olive and jojoba oils, the activation entropy is less negative, indicating a higher degree of disorder relative to sunflower and rosehip oils. This suggests a higher probability of forming the activated complex, owing to less restrictive spatial orientations for electron transfer. Consequently, these oils exhibit greater antioxidant capacity, although jojoba shows marked temperature-dependent variations.

Table 3 shows that the activation energy (∆G^#^) values for all oils are relatively similar (~56–58 kJ/mol), indicating that the reactions have comparable overall viability. The ΔH^#^ results are positive, signifying that the reactions of oxidation process are endothermic. These results affirm previous studies, which have demonstrated that the oxidation process is endothermic for several refined oils, including sunflower, sesame, and grape oils [43]. However, differences in activation enthalpy (ΔH^#^) and activation entropy (ΔS^#^) determine how each system reaches the transition state.

Among the oils, jojoba oil exhibits the highest ΔH^#^ and ΔG^#^ values, implying that it requires more energy to reach the transition state. In contrast, sunflower and rosehip oils have lower energy barriers. Specifically, rosehip oil shows an intermediate ΔH^#^ value and a higher rate value constant (k) than jojoba oil at 298 K, while sunflower oil displays the lowest ΔH^#^ and a high k at 298 K. This may be related to the composition, as it contains many tocopherols, which are antioxidants [27].

The results for jojoba oil suggest a change in kinetic regime above 340 K. Its relatively high ΔH^#^ indicates a larger enthalpic barrier to reach the transition state, while its less negative ΔS^#^ implies a smaller entropic penalty for activated-complex formation. Together with the strong exponential temperature dependence of TAC, and the marked increase in the rate of TAC change with temperature above 340 K, this behavior may suggest that a physicochemical change or a shift in the dominant oxidation mechanism facilitates transition-state formation and increases the reaction rate. In terms of ΔG^#^ = ΔH^#^ − TΔS^#^, the process appears more enthalpy-controlled below 340 K, whereas the temperature-dependent entropic contribution becomes more influential above 340 K.

This interpretation is consistent with the rate constants determined at 298 K, where jojoba oil exhibits the lowest value among all oils studied, and with the constant measured at 373 K, which becomes the highest. This behavior may be attributed to the particular composition of jojoba oil, characterized by a very high proportion of MUFAs (97.0%) and a very low proportion of PUFAs (1.0%, Table 1). MUFAs are substantially more resistant to oxidation than PUFAs, which would account for the behavior observed up to 340 K if MUFAs dominate the reactivity in this temperature range. If, above this temperature, the fraction of MUFAs undergoing oxidation increases, this would lead to the formation of larger amounts of hydroperoxides, which could in turn enhance the overall reactivity of the system by initiating chain reactions.

Olive oil exhibits a higher ΔH^#^ than sunflower oil but still shows a relatively high rate constant at 373 K. According to the Eyring model, the rate constant depends on both activation enthalpy and activation entropy, and therefore the combined contribution of these parameters determines the observed kinetic behavior. The presence of bioactive compounds in olive oil, such as hydroxytyrosol, which has been reported to possess strong antioxidant activity [44], may also contribute to this behavior. Moreover, olive oil exhibits the second-highest ΔH^#^ and the second-least-negative ΔS^#^ after jojoba oil, suggesting a similar temperature-dependent behavior. This interpretation is consistent with the trend observed in the corresponding plot: at 298 K, the antioxidant capacity of olive oil is lower than that of sunflower oil, whereas above 300 K its TAC values become the highest among the oils compared (Figure 2). It is noteworthy that olive oil contains a higher proportion of MUFAs (69.0%) than sunflower (27.0%) and rosehip oil (20.4%), although still lower than that of jojoba oil (97.0%). Furthermore, olive oil contains nearly ten times more PUFAs (9.2%) than jojoba oil (1.0%) (Table 1).

With respect to sunflower and rosehip oils, both exhibit activation enthalpies that are very similar to each other, and appreciably lower than those of jojoba and olive oils. The same applies to their activation entropies, which are more negative compared with jojoba and olive oils and also very similar to each other (Table 3). The plot shows that the temperature dependence of their antioxidant capacities evolves in parallel (Figure 2). It is worth noting that the MUFA and PUFA compositions of these two oils are quite similar (MUFA/PUFA ratio are 0.45 and 0.28 respectively), as shown in Table 1, with a much higher PUFA content than olive and jojoba oils. The MUFA/PUFA ratios of olive and jojoba are 7.5 and 97.0 respectively.

**Figure 3:**
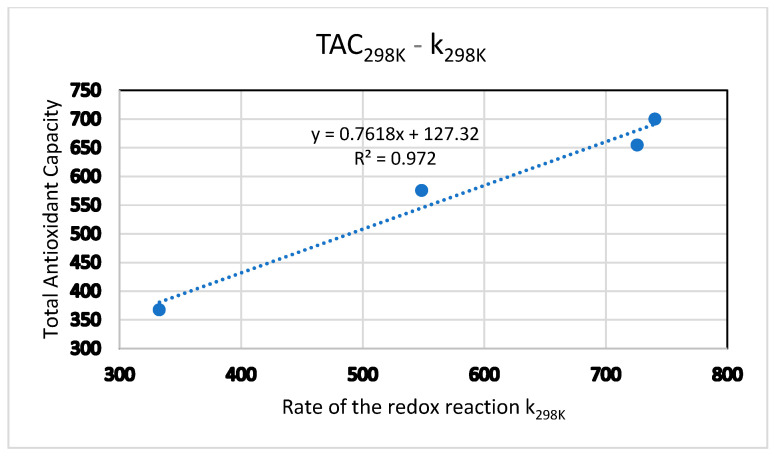
Relationship between TAC and k (s^−1^) at 298 K.

Figure 3 shows the lineal relationship of TAC and k (s^−1^), both at 298 K. It can be seen that the higher the total antioxidant capacity (TAC), the higher the k_298K_ value of the redox reaction.

### 3.3. Assessment of Oxidative Stability Using the Rancimat Method

The stability of vegetable oils is related to their UFA content, particularly the amount of PUFAs, which are more susceptible to oxidation than MUFAs because they have more double bonds. Table 4 summarizes the induction period (IP) at 373 K obtained using the Rancimat method for sunflower, olive, jojoba and rosehip oils in their pure form.

Among the oils evaluated, olive and jojoba oils showed the highest oxidative stability, followed by sunflower oil and, finally, rosehip oil (Table 4). This trend is related to their fatty acid composition.

Jojoba oil presents the highest oxidative stability and is a peculiar oil, mainly due to its unique chemical structure [45]. Unlike most vegetable oils, which are composed primarily of triglycerides, jojoba oil is actually a liquid wax ester made up of long-chain monounsaturated fatty acids and alcohols. The ester bonds and long, straight hydrocarbon chains make it much more resistant to oxidation compared to triglycerides. Moreover, jojoba oil has a low degree of unsaturation, with very few polyunsaturated fatty acids and mostly monounsaturated long-chain wax esters. The proportion of polyunsaturated components is extremely low (Table 1), which is one of the reasons why the oil is so chemically stable and resistant to oxidation.

Regarding the induction period, jojoba oil exhibits the longest induction time, indicating the greatest resistance to oxidation under the Rancimat conditions. It should be noted that the Rancimat test reflects oxidative stability under accelerated air/oxygen exposure at elevated temperature and is especially sensitive to the formation of secondary oxidation products. Since MUFAs generally oxidize more slowly than PUFAs, the high MUFA (97%) content and very low PUFA (1%) fraction of jojoba oil can account for its long induction period. This observation does not contradict the BRS results (Figure 2), because the two experiments operate on substantially different timescales and in different media. In particular, the BRS emulsifies the oil in an aqueous electrolyte/methanol-containing phase, which can promote oxidation indirectly by altering interfacial properties and mass transfer (and potentially through catalytic effects if pro-oxidant species are present). The formation of microdroplets or nano-emulsions increases interfacial area and can enhance reactive encounters among species, making such systems more sensitive to temperature than bulk oil.

Olive oil is rich in MUFAs (69.0%), which gives it greater resistance to oxidation. Sunflower oil has an intermediate composition, with 27.0% MUFAs and 60.0% PUFAs (Table 1), giving it medium oxidative stability, while rosehip oil has a high content of PUFAs (72.0%), making it more susceptible to oxidation (Table 1).

The results obtained are consistent with those of previous studies [46,47], confirming the well-established relationship between fatty acid composition and oxidative stability. Yurchenko and Saealle [48] reported considerable variability in fatty acid profiles and induction periods, highlighting the strong influence of fatty acid type on oxidative stability. In agreement with these findings, the present study confirms that oils rich in monounsaturated fatty acids (MUFAs), such as extra virgin olive oil, exhibit greater oxidative stability and are therefore more suitable for applications requiring extended shelf life.

Previous research conducted in Egypt [49] evaluated the oxidative stability of several oils using the Rancimat method and reported the lowest induction period in sunflower oil, attributed to its high polyunsaturated fatty acid (PUFA) content, particularly linoleic acid, and its greater degree of unsaturation. In contrast, extra virgin olive oil showed the highest oxidative stability due to its higher monounsaturated fatty acid (MUFA) content, mainly oleic acid, which oxidizes much more slowly than linoleic acid.

The Rancimat test provides an overall estimate of the antioxidant capacity of oils but does not allow for identification of the contribution of individual compounds or their potential interactions. Therefore, oxidative stability does not depend on a single factor but is determined by fatty acid composition together with a complex pool of endogenous antioxidant compounds. Both factors interact in complex ways and jointly influence the oxidative stability of edible and cosmetic vegetable oils [50].

### 3.4. Relationship Between the Results of the BRS Test and the Rancimat Method

#### 3.4.1. Principal Component Analysis (PCA) and Multivariant Analysis of Composition and Properties of Oils

The PCA was performed using the following variables: the transition state properties of oil oxidation with the BRS electrolyte k (s^−1^) and ∆G^#^ at 298 K, ∆H^#^, ∆S^#^ (Table 3), the induction period (IP) (Table 4), TAC values at 298 K (Figure 1), and the composition of oils in terms of MUFAs, PUFAs, UFAs, and SFAs (Table 1), together with MUFA/PUFA, SFA/MUFA, SFA/UFA and SFA/PUFA ratios.

**Figure 4:**
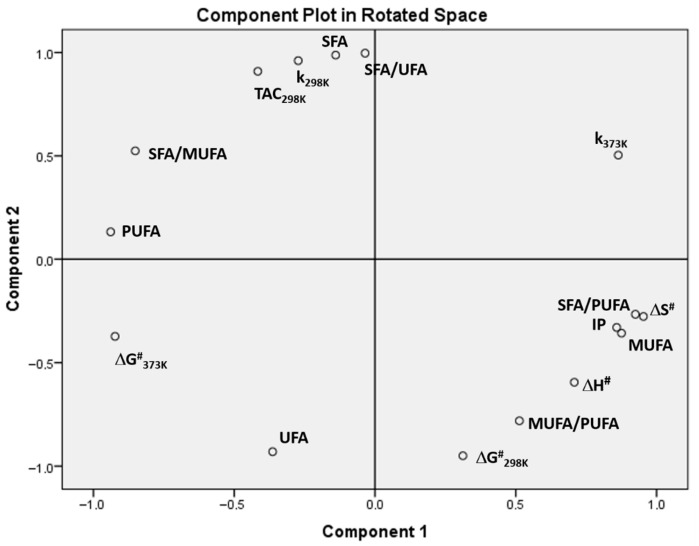
Score plot of variables or properties of oils. SFA/MUFA: SFA/MUFA ratio; SFA/PUFA: SFA/PUFA ratio; SFA/UFA: SFA/UFA ratio and MUFA/PUFA: MUFA/PUFA ratio.

The PCA allowed the original information from all variables to be reduced to two main components: component 1, which explains 94.45% of the variance, and component 2, which represents 5.49%, adding up to a total of 99.94% of variance. Figure 4 shows the score plot with the results of the PCA.

Figure 4 shows the score plot graph, where component 1 > 0 with greater importance in percentage of variance (94.45%) is observed as the variables associated with greater oxidative stability such as IP, MUFAs, ∆G^#^ at 298 K, k at 373 K, and ∆H^#^, and the SFA/PUFA and MUFA/PUFA ratios correlate positively. This means that the IP increases with the presence of a higher degree of SFAs, followed by MUFAs, and decreases with the presence of PUFAs, as already known from previous studies [20]. On the other hand, the variables referring to the transition state, ΔH^#^ values, imply that it requires more energy to reach the transition state and therefore indicate lower oxidant efficiency and, consequently, higher IP.

In contrast, variables such as PUFAs and UFAs appear on the opposite side of the graph, component 1 < 0, so there is a negative correlation with IP and therefore their increase diminishes oxidative stability and IP.

It is important to note that oxidative stability and IP correlate positively with ∆G^#^ at 298 K and k at 373 K and negatively with ∆G^#^ at 373 K and k at 298 K, which is consistent with oxidative stability and IP decreasing with increasing temperature [51]. Although we see that TAC values at 298 K in about 5 s correlate negatively with IP, it should be noted that Rancimat gives IP values at 373 K, in longer periods, so they cannot be related.

The PCA provided a coherent visualization of the relationships among variables, showing patterns consistent with the correlation and regression analyses. In particular, the separation of samples reflected the expected behavior of lipid systems, where oils with higher PUFA content exhibited lower oxidative stability, while MUFA-rich samples showed greater resistance to oxidation.

Although PCA results should be interpreted as exploratory due to the limited sample size, the observed trends are chemically meaningful and consistent with established mechanisms of lipid oxidation.

#### 3.4.2. Analysis of Composition and Properties of Oils

The analysis using the same variables as for PCA confirmed the results previously obtained with the BRS and Rancimat methods.

**Figure 5:**
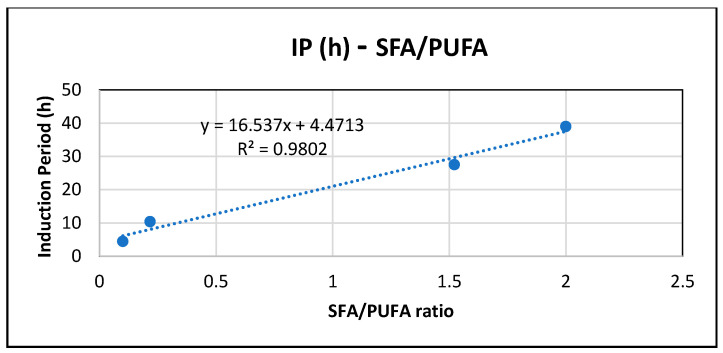
Relationship of IP (h) at 373 K with SFA/PUFA ratio for oil samples.

Figure 5 shows the relation of IP (h) at 373 K with the SFA/PUFA ratio for each oil. The results confirm that oxidative stability, and therefore the induction period (IP), increase as the percentage of saturated fatty acids (%SFA) increases and the percentage of polyunsaturated fatty acids (%PUFA) decreases. This confirms that oxidative stability increases as the degree of unsaturation of the oil decreases. The result is consistent with PCA (Figure 4), where IP and SFA/PUFA ratio are found in the same quadrant (component 1 > 0 and component 2 > 0), and with previous results [20], where it has been suggested that oils rich in PUFAs, such as rosehip oil, with the lowest SFA/PUFA ratio (0.10 in Table 1), are more prone to oxidative degradation due to their higher degree of unsaturation.

**Figure 6:**
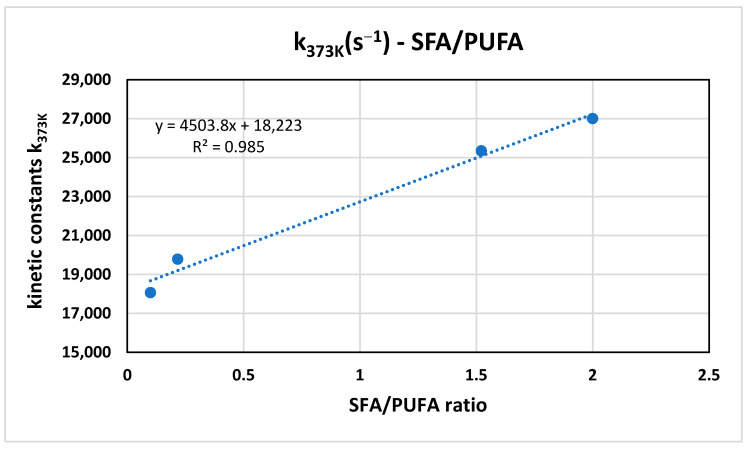
Relationship of kinetic constant at 373 K with SFA/PUFA ratio for oil samples.

We also highlight the importance of the relationship between the kinetic constant of the redox reaction and the SFA/PUFA ratio (Figure 6). It can be seen that the higher the SFA/PUFA ratio, (i.e., the lower the degree of unsaturation of the oil), the higher the k_373K_ value of the redox reaction between the antioxidants in the oil and the BRS electrolyte. This result is also confirmed in the PCA (Figure 4), where the SFA/PUFA ratio and k_373K_ have a positive correlation (component 1 > 0; 94.45% variance).

#### 3.4.3. Relation Between IP (Rancimat) and Rate Constant k Calculated with Data TAC from BRS Test

Comparing BRS-Redox and Rancimat showed that the effectiveness of both systems is influenced by the experimental conditions, demonstrating that the oxidative stability of the same oil can vary depending on the parameters considered. BRS-Redox is applied for short periods of time (about 4–5 s) at different temperatures (298–373 K), while Rancimat acts at high temperatures (373 K) for long periods (h) until oxidation products appear.

**Figure 7:**
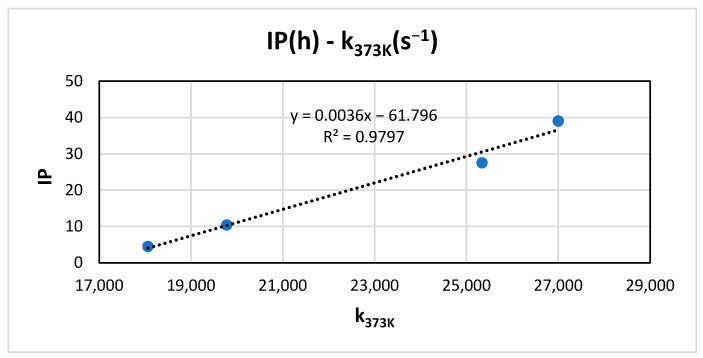
Relationship of kinetic constant of the redox reaction and induction period at 373 K for each oil sample.

Recalling that the IP was measured at 373 K and that the kinetic constants of the redox reaction were calculated at 373 K using the Arrhenius equation, a linear relationship between them can be observed (Figure 7). In PCA, a positive correlation is observed between IP (component 1 > 0; 94.45% variance) and k_373K_ (Figure 4), which shows consistency between the results obtained from the BRS test and the Rancimat method.

The apparent lack of direct correspondence between the Rancimat induction period (IP) and the antioxidant activity measured by the BRS method arises from the fact that both techniques probe related but not equivalent aspects of oxidation. The Rancimat IP reflects the overall oxidative stability of a complex lipid system under accelerated conditions, integrating factors such as lipid composition, antioxidant content, and propagation reactions. In contrast, the TAC-derived kinetic constant (k) obtained by BRS represents the intrinsic radical scavenging capacity under simplified and controlled conditions of temperature, focusing on the reaction rate with specific radicals.

Notwithstanding these differences, our results show a strong correlation between IP and k, indicating that compounds with higher radical scavenging rates effectively translate into enhanced oxidative stability. This finding supports a direct mechanistic link between intrinsic antioxidant reactivity and macroscopic oxidation resistance. The excellent agreement observed reinforces the relevance of combining both approaches and highlights the novelty of this work, as it bridges molecular-level kinetic parameters with bulk oxidation behavior in lipid systems. Therefore, rather than being redundant, the two methods provide complementary and synergistic information, offering a more comprehensive understanding of antioxidant performance.

#### 3.4.4. Study Considerations

The present study provides valuable insights into the oxidative stability and antioxidant behavior of vegetable oils; however, certain limitations should be considered. Due to the limited sample size (n = 4), principal component analysis (PCA) was employed only as an exploratory tool. Standard adequacy tests, such as the Kaiser–Meyer–Olkin (KMO) measure and Bartlett’s Test of Sphericity, were not considered reliable under these conditions, and therefore the PCA results should be interpreted in a descriptive manner.

In addition, the BQC-Redox System (BRS) represents a relatively recent analytical technique that has not previously been applied to the evaluation of antioxidant capacity in vegetable oils. Although previous studies have demonstrated that this method provides precise and reproducible measurements, the available scientific literature on its application in lipid systems remains limited. This restricts direct comparison of the results obtained in this study with previously reported data. Furthermore, another consideration of this study is that, in the BRS test, we worked with emulsions, and variability in the measurements was occasionally observed because the distinct phases (oil/methanol/electrolyte) must be maintained, which requires working quickly. We believe that the use of emulsifying agents could affect electron transport in the electrolyte if the procedure is not carried out with great precision and care.

However, despite this, the average values obtained in the BRS test are consistent with the behavior of the oils and with the Rancimat results, demonstrating the reliability of both methods and the potential applicability of these techniques for assessing the oxidative stability of vegetable oils.

Another consideration is that the role of the individual antioxidant compounds in each one of the oils used, as well as their potential interactions in determining oxidative stability, is not known.

Despite these limitations, the results highlight the importance of selecting vegetable oils with well-characterized compositions according to their intended applications, whether in cosmetic or food systems. Consequently, future research should focus on these aspects.

## 4. Conclusions

- Overall, increasing temperature enhanced antioxidant activity (TAC) in all oils. Total antioxidant capacity (TAC) of olive, sunflower, and rosehip oils increased linearly with temperature; olive oil showed the strongest temperature dependence, as indicated by the highest slope of the regression lines. In addition, the k_373_/k_298_ ratios were consistent with the slopes of the regression lines.
- Jojoba oil exhibited an exponential increase in TAC with temperature. Below 340 K, jojoba oil had lower TAC than other oils; above 340 K, TAC increased rapidly, giving much higher k_373_/k_298_ values (26.71–34.92). At 373 K, jojoba oil showed a markedly accelerated oxidation process (k_373_/k_298_ = 81.16).
- Under accelerated oxidation (373 K), oxidative stability (Rancimat, IP) ranked as follows: jojoba > olive > sunflower > rosehip oil. This trend correlates with degree of unsaturation; lower unsaturation leads to higher stability. MUFA/PUFA ratios (97.0, 7.5, 0.45, 0.21) support the observed stability order.
- Although the Rancimat induction period and the BRS-derived kinetic constant evaluate different aspects of oxidation, both methods showed a strong correlation, demonstrating that higher intrinsic radical scavenging activity is effectively associated with improved oxidative stability in lipid systems. While the Rancimat method reflects the global behavior of complex lipid matrices under accelerated oxidation conditions, the BRS approach provides molecular-level information on antioxidant reactivity under controlled conditions. The excellent agreement between both techniques highlights their complementary nature, providing a more comprehensive understanding of antioxidant performance. Moreover, these findings establish a direct mechanistic relationship between antioxidant kinetics and macroscopic oxidation resistance, underscoring the novelty and relevance of combining both methodologies for the evaluation of antioxidants in vegetable oils.
- These findings are relevant for researchers, food technologists, consumers, and regulatory bodies. The results support the informed selection of oils with better quality and shelf life. This study highlights the importance of selecting well-characterized vegetable oils depending on their application (food or cosmetics). Future research should focus on the role and interactions of individual antioxidant compounds in oxidative stability.

## Figures and Tables

**Table 1:** Fatty acid composition of the analyzed oil samples as provided by the manufacturers.

Oil	SFA (%)	MUFA (%)	PUFA (%)	UFA (%)	MUFA/PUFA	SFA/PUFA
**Sunflow** **er**	13.0	27.0	60.0	87.0	0.45	0.22
**Olive**	14.0	69.0	9.2	78.8	7.50	1.52
**Rosehip**	7.2	20.4	72.0	92.4	0.28	0.10
**Jojoba**	2.0	97.0	1.0	94.0	97.00	2.00

SFA: Saturated Fatty Acids; UFA: Unsaturated Fatty Acids; MUFA: Monounsaturated Fatty Acids; PUFA: Polyunsaturated Fatty Acids.

**Table 2:** Equations of TAC of oils vs. temperature (K).

Oil Sample	Equation	R^2^
**Sunflower**	y = 11.476x − 2694.5	0.9591
**Olive**	y = 26.095x − 7123.4	0.9858
**Rosehip**	y = 11.261x − 2816.5	0.9757
**Jojoba**	y = 0.6062 e^0.0215x^	0.9842

**Table 3:** Thermodynamic and kinetic parameters calculated from the Eyring equation for pure rosehip, jojoba, sunflower and olive oil samples based on the TAC values obtained using the BRS test.

Oil Sample	ln (TAC/T) = *f* (1/T)	ΔH^#^ (kJ/mol^−1^)	ΔS^#^ (kJ/mol^−1^ K^−1^)	ΔG^#^ (kJ/mol^−1^)	k (s^−1^) at 298 K	k (s^−1^) ^1^ at 373 K
**Sunflower**	y = −829.54x + 3.6947	6.90	−166.89	56.60	740.40	19,778
**Olive**	y = −1688.6x + 6.5657	14.04	−143.05	56.67	725.73	25,339
**Rosehip**	y = −1045.0x + 4.1423	8.60	−163.36	57.39	548.50	18,064
**Jojoba**	y = −2129.0x + 7.2643	17.70	−137.20	58.60	332.68	26,999

^1^ Calculated from k (s^−1^) at 298 K by Arrhenius equation. ΔH^#^: activation enthalpy, ΔS^#^: activation entropy, ΔG^#^: activation Gibbs free energy, k: kinetic constant.

**Table 4:** Induction time of samples of oils using the Rancimat method.

Oil Sample	Induction Period (Hours)
Sunflower	10.39 ± 0.03
Olive	27.50 ± 2.08
Rosehip	4.45 ± 0.07
Jojoba	39.80 ± 3.46

## Data Availability

The original contributions presented in this study are included in the article. Further inquiries can be directed to the corresponding authors.
